# Putting Within-Country Political Differences in (Global) Perspective

**DOI:** 10.1371/journal.pone.0231794

**Published:** 2020-04-23

**Authors:** Ximena Garcia-Rada, Michael I. Norton

**Affiliations:** Marketing unit, Harvard Business School, Boston, MA, United States of America; Carnegie Mellon Univeristy, UNITED STATES

## Abstract

The current political discourse in the United States focuses on extreme political polarization as a contributor to ills ranging from government shutdowns to awkward family holidays. And indeed, a large body of research has documented differences between liberals and conservatives–primarily focused on Republicans and Democrats in the United States. We combine large international surveys and more fine-grained surveys of United States citizens to compare differences in opinion between Republicans and Democrats to the full range of world opinion on moral issues (*N* = 37,653 in 39 countries) and issues of free speech (*N* = 40,786 in 38 countries). When viewed in the full distribution, polarization between Democrats and Republicans appears relatively small, even on divisive issues such as abortion, sexual preference, and freedom of religious speech. The average Democrat-Republic overlap is greater than 70% of the country pair overlaps across eight moral issues, meaning that 70% of the country pairs are more dissimilar from each other than Democrats and Republicans are dissimilar; similarly, the average Democrat-Republic overlap is greater than 79% of the country pair overlaps across five freedom of speech issues. These results suggest that cross-cultural comparisons are useful for putting differences between political partisans within the same country in context.

## Introduction

Heated discussions on social issues such as abortion, gay marriage, and free speech have characterized politics in the United States for many years; political leaders have suggested and scholars have provided evidence of a steady increase in the gap between opinions of Democratic and Republican citizens on a host of issues [1–3]. Moreover, polarization has been linked to a number of troubling outcomes, from decreased voter turnout to inequality to the increased dominance of single-issue interest groups on the political process [4]. We argue that dissimilarities between Democrats and Republicans may mask some underlying level of agreement, such that disagreements between Republicans and Democrats may be less extreme than they are often perceived. We suggest that placing the views of both parties in global perspective–comparing them not only to each other but to citizens of nearly 40 countries–demonstrates that political partisans in the United States exhibit markedly more similar views to each other than to citizens of other countries.

Certainly, decades of research have documented robust differences between liberals and conservatives on a host of psychological dimensions. Some research grounds the dissimilarities between the two groups in personality traits [5]: liberals are more open to experience and think more analytically, whereas conservatives prefer familiarity, stability, and predictability [6, 7]. Carney and colleagues [8] offer a theoretical framework and empirical evidence to support the notion that liberals score higher in openness to experience–pursuing creativity, novelty, and diversity–while conservatives score higher in conscientiousness and prefer lives that are orderly, conventional, and organized. Research in neuroscience has assessed differences in neurocognitive functioning of liberals and conservatives [9]. And research in moral psychology suggests that liberals and conservatives have different moral values and vary in the moral foundations most central to their judgments and decisions [10]: liberals rely primarily on two foundations (harm/care and fairness/reciprocity), whereas conservatives rely on all five dimensions [11]. Indeed, the number of investigations demonstrating additional differences between the two groups is too large for this brief review, ranging from different moral intuitions to different sensitivity to deviance [12, 13].

Despite these many differences, scholars have noted that the differences between the two groups are small in magnitude and identified underlying similarities. For example, Carney and colleagues [8] concluded that “although our studies show clearly that there are genuine differences between liberals and conservatives, we do not wish to overstate the magnitude or significance of these differences (pp. 835).” Similarly, psychologists have noted that although the groups differ on which moral foundations they draw on most heavily, both draw on all five foundations [14–16]. Schein and Gray [17] suggest further similarities in moral judgment between liberals and conservatives, and Frimer, Skitka, and Motyl [18] demonstrate that individuals from both groups are similarly motivated to avoid crosscutting information. In addition, an emerging body of literature demonstrates that while liberals and conservatives differ in their ideal levels of wealth and income inequality, for both groups, their ideal levels are more equal than their estimates of the current level of inequality [19, 20].

However, even when differences are small, research suggests that people can continue to demonstrate false polarization in their perceptions of differences–believing that they are “lone moderates” but others are more extreme [21, 22]; indeed, Graham, Nosek and Haidt [23] demonstrate that while people are accurate in guessing the direction of differences between liberals and conservatives, they overestimate these differences significantly. Similarly, Westfall et al. [24] use national survey data from the American National Election Study to show that people in the United States overestimate polarization between attitudes of Democrats and Republicans. Overestimating differences between groups has troubling implications: it reduces liking and cooperativeness toward outgroup members [25] and drives negative out-group attributions in competitive contexts [26] as well as ingroup favoritism [27].

We provide a novel approach to reframing polarization between liberals and conservatives, even on divisive issues–including abortion, homosexuality, and freedom of religious expression–by placing any differences that do exist in the context of the full range of opinions in countries across the world. We suggest that cross-cultural comparisons offer a unique lens into understanding the relative magnitude of the differences between Republicans and Democrats, demonstrating that, placed in context, their similarities may be more pronounced than their differences.

## Methods and results

### Dataset #1: Moral issues

We first compare levels of moral acceptability for eight moral issues across 39 countries. We used data from a survey conducted by the Pew Research Center in Spring 2013 [28] among adult populations, in native languages and using telephone and face-to-face interviews (37,653 respondents from 39 countries; *M*_*age*_ = 41.72, *SD*_*age*_ = 16.49; 50.8% female). For each of eight moral issues–Abortion, Alcohol Use, Contraception Use, Divorce, Extramarital Affairs, Gambling, Homosexuality, and Premarital Sex–survey respondents answered: “Do you personally believe that [this issue] is morally acceptable, morally unacceptable, or is not a moral issue?” Response options were “morally acceptable,” “morally unacceptable,” “not a moral issue,” “depends on the situation,” and “don’t know”; participants could also refuse to answer each question (see S1 Appendix for exact items and response options).

**Fig 1:**
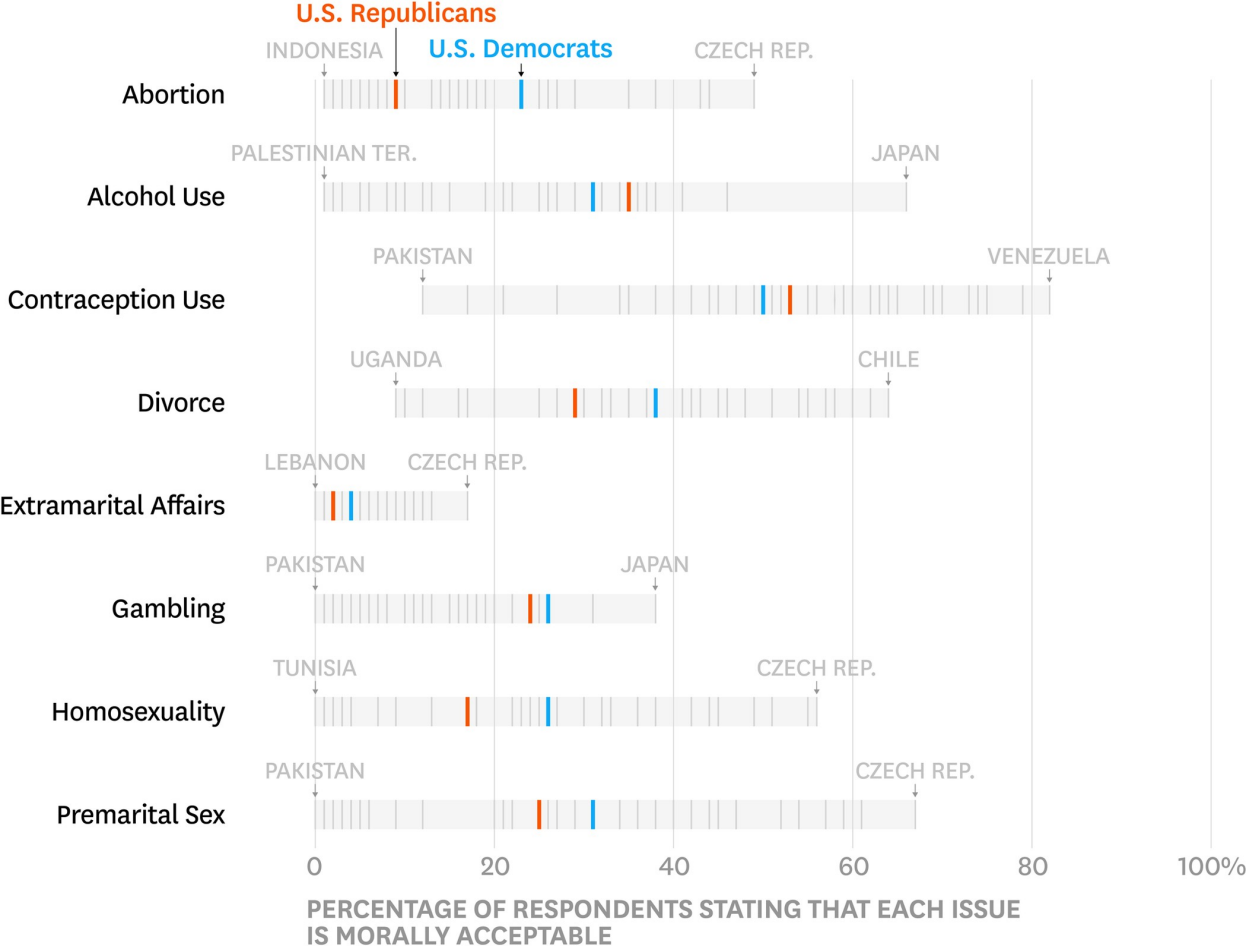
Moral issues: Democrats and republicans in global context.

The United States sample included 1,002 respondents: Democrats (*n* = 328), Independents (*n* = 343), and Republicans (*n* = 259); 72 respondents were recorded as no preference, other party, don’t know, or refused. Fig 1 presents results descriptively: we placed Democrats and Republicans in the global distribution by plotting the proportion of respondents in each country and also within Democrats and Republicans in the United States who indicated each issue was “morally acceptable.” For full distributions of all responses in the United States, see S1 Table.

To measure similarities between two groups, we adapted the overlapping coefficient approach [29–31]. Because we had nominal rather than continuous measures, we calculated index of dissimilarities as the ratio of the number that must be moved from cells of excess to cells of deficit to achieve even distributions [32]. To illustrate, imagine that five Democrats and five Republicans answered a question with only two response options: yes and no. If two Democrats selected *yes* (and three selected *no*) and three Republicans selected *yes* (and two selected *no*), changing just one out of the five responses from the Republican group from “yes” to “no” would produce identical distributions. In this example, the overlap coefficient between Democrats and Republicans would be 80% (= 4/5) and the index of dissimilarity would be 20% (= 1/5).

**Fig 2:**
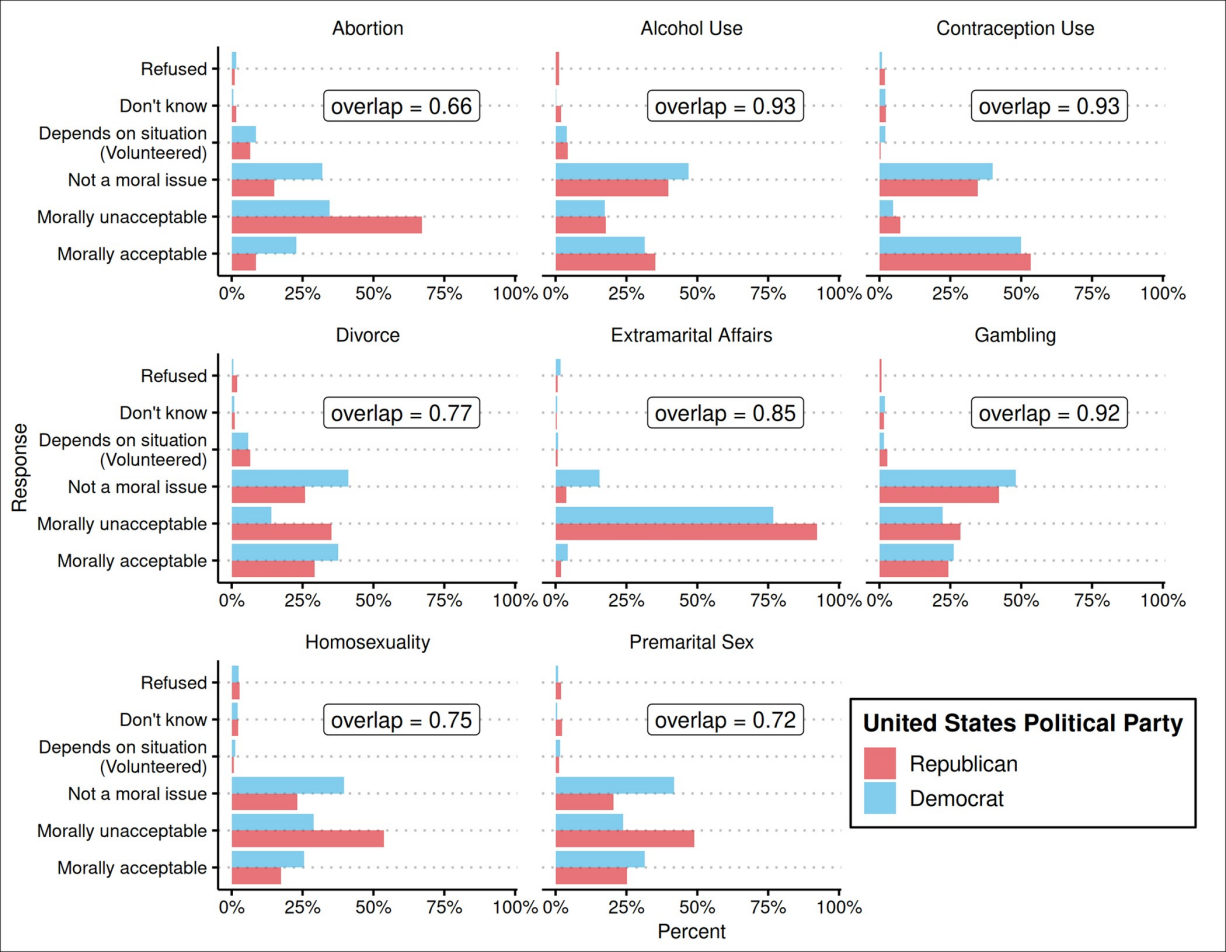
Democrat-Republican overlap for moral issues.

**Fig 3:**
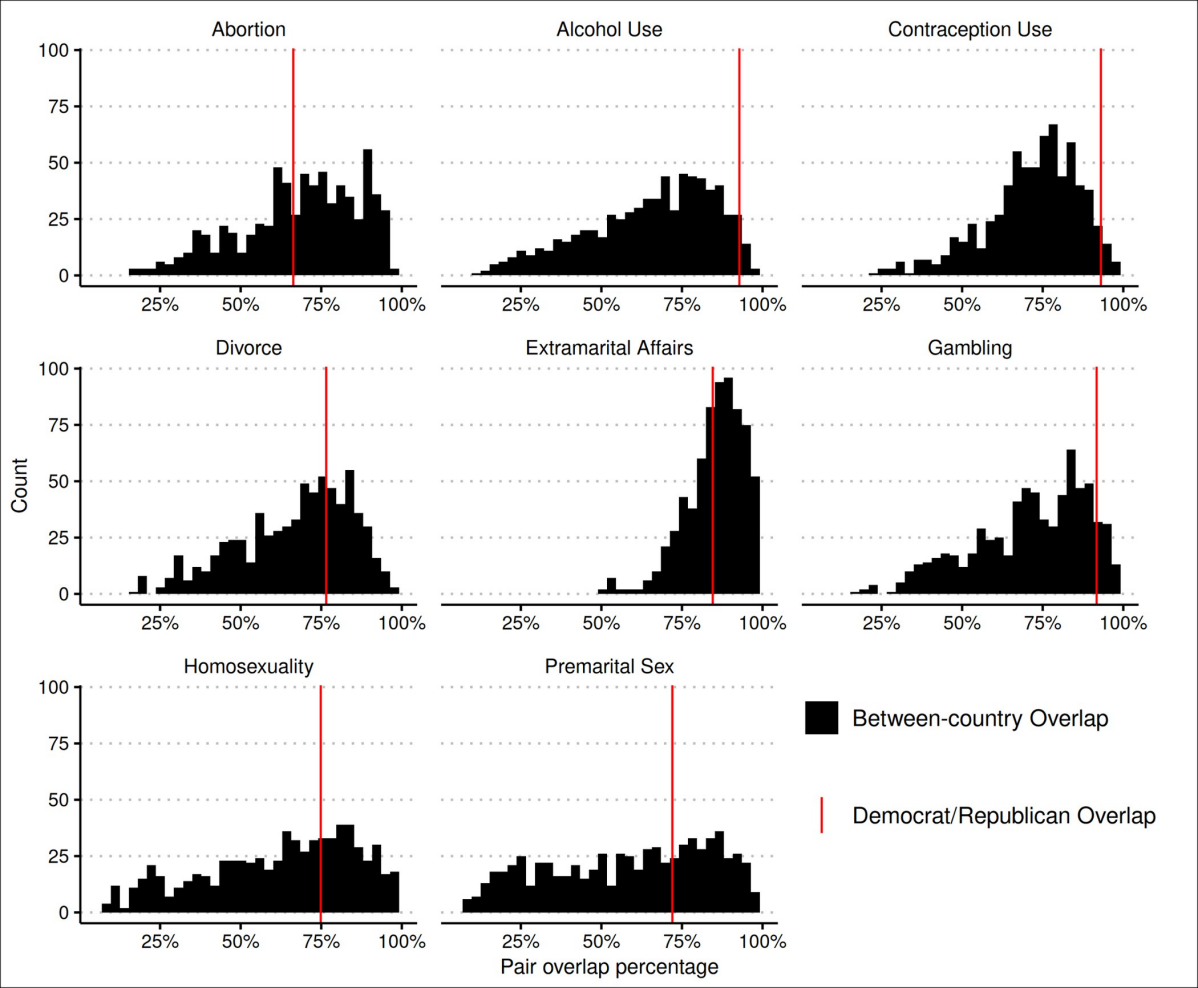
Distribution of all country pair overlap for moral issues.

We used this procedure to calculate a Democrat-Republican overlap for each moral issue using the StatMatch R package [33], and computed 95% confidence intervals using a bootstrap procedure (see Democrat-Republican Overlap for eight moral issues in Fig 2); for ease of interpretation, we used one minus the index of dissimilarity as the measure of overlap. Using the same procedure, we calculated overlap coefficients for all country pairs and plotted the distribution of these overlap coefficients (see Fig 3). Finally, for each moral issue, we calculated the percentage of country pair overlaps that were smaller than the Democrat-Republican overlap, along with bootstrapped 95% confidence intervals for this statistic (see Table 1).

**Table 1:** Overlap coefficients for moral issues.

	Democrat-Republican Overlap			The Democrat-Republican overlap is greater than X% of the country pairs		
Moral Issues	Overlap Coefficient	95% Confidence Interval of Overlap Coefficient		X Statistic	95% Confidence Interval of X Statistic	
Abortion	66%	60%	74%	42%	28%	58%
Alcohol Use	93%	89%	100%	97%	94%	100%
Contraception Use	93%	89%	100%	97%	94%	100%
Divorce	77%	70%	84%	66%	51%	85%
Extramarital Affairs	85%	80%	91%	40%	16%	58%
Gambling	92%	87%	100%	90%	83%	100%
Homosexuality	75%	68%	83%	65%	53%	79%
Premarital Sex	72%	65%	80%	63%	52%	73%

As in previous research, Democrats and Republicans in the United States did not fully agree: while the Democrat-Republican overlap coefficients for all moral issues are at 66% or above (with Abortion being the most “polarizing” issue), the average overlap across issues was 81% and no issue showed 100% overlap.

However, putting Democrat-Republican disagreement in global context revealed that, across the eight issues, the average Democrat-Republic overlap is greater than 70% of the country pair overlaps, meaning that 70% of the country pairs are more dissimilar from each other than Democrats and Republicans are dissimilar form each other. For six of the moral issues, the Democrat-Republic overlap was greater than more than half of the country pair overlaps as the confidence interval for those statistics excluded 50% (columns 5–6 in Table 1). And for the two issues with the largest disagreement, the Democrat-Republican overlap was still greater than 42% of all country pairs (Abortion) and 40% than all country pairs (Extramarital Affairs).

### Dataset #2: Freedom of speech issues

For these eight moral issues, Americans are often in the middle of the world distribution. Do Republicans and Democrats agree with each other more than they do with citizens of other countries simply because they have more “middling” opinions? To address this issue, we next compare levels of agreement on questions regarding freedom of expression across 38 countries using a second dataset. The Pew Global Attitudes Survey was administered in Spring 2015 (40,786 respondents from 38 countries; *M*_*age*_ = 41.92, *SD*_*age*_ = 16.92; 50.6% female) [34]. Respondents answered five questions about freedom of expression statements: Call for Violent Protests, Criticize the Government’s Policies, Offensive to Minorities, Offensive to Your Religion and Beliefs, and Sexually Explicit. Response options were “People should be able to say these things publically,” “Government should be able to prevent people from saying these things,” and “Don’t know”; participants could also refuse to answer each question (see S2 Appendix for exact items and response options).

**Fig 4:**
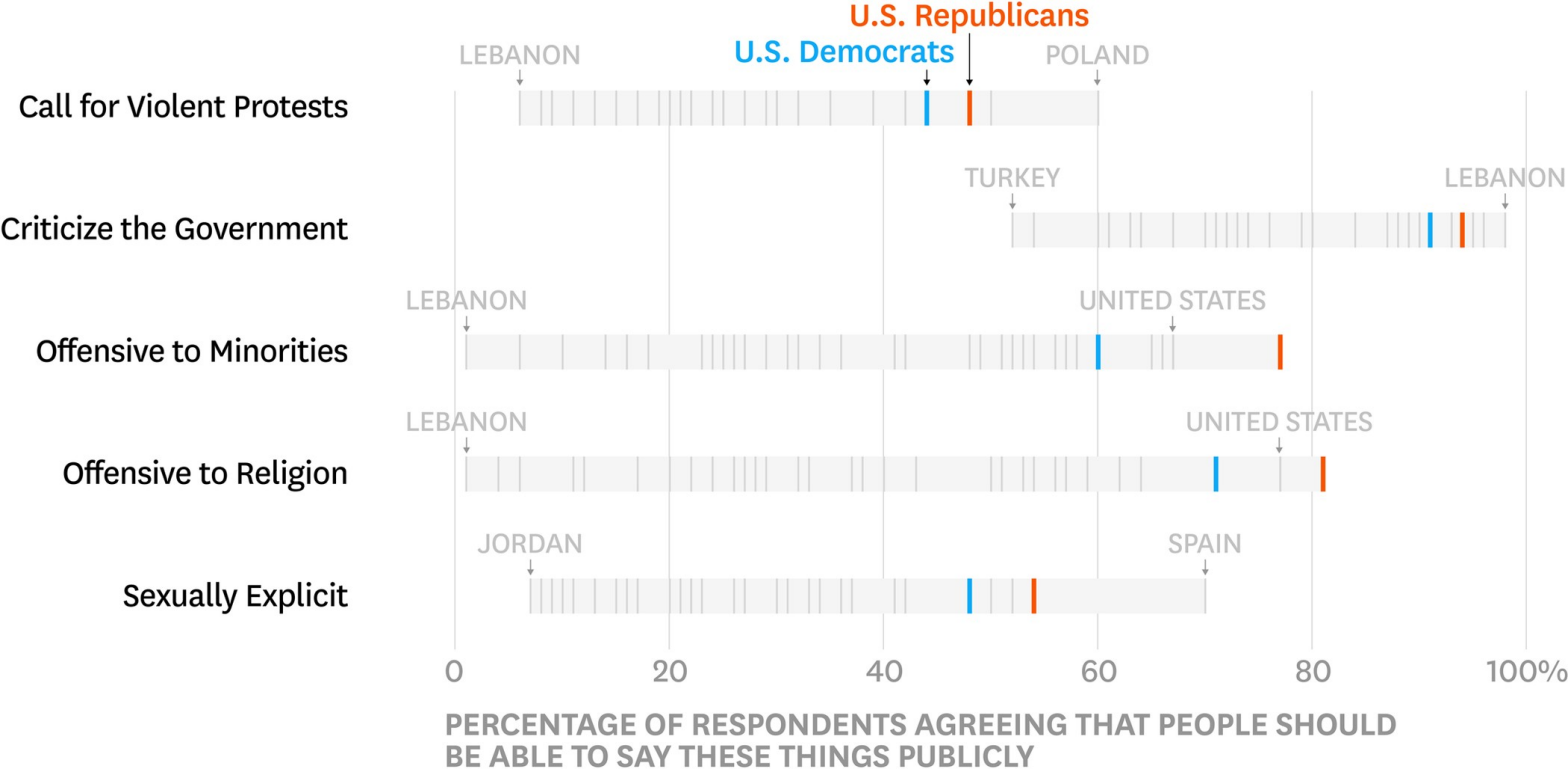
Freedom of speech issues: Democrats and republicans in global context.

The United States sample included 1,003 respondents: Democrats (*n* = 291), Independents (*n* = 365), and Republicans (*n* = 286); 61 respondents were recorded as no preference, other party, don’t know, or refused. Fig 4 presents results descriptively: we first put Democrats and Republicans in global context by plotting the proportion of respondents in each country and also within Democrats and Republicans in the United States who indicated “People should be able to say these things publically” for each issue. For full distributions of responses in the United States, see S2 Table.

**Fig 5:**
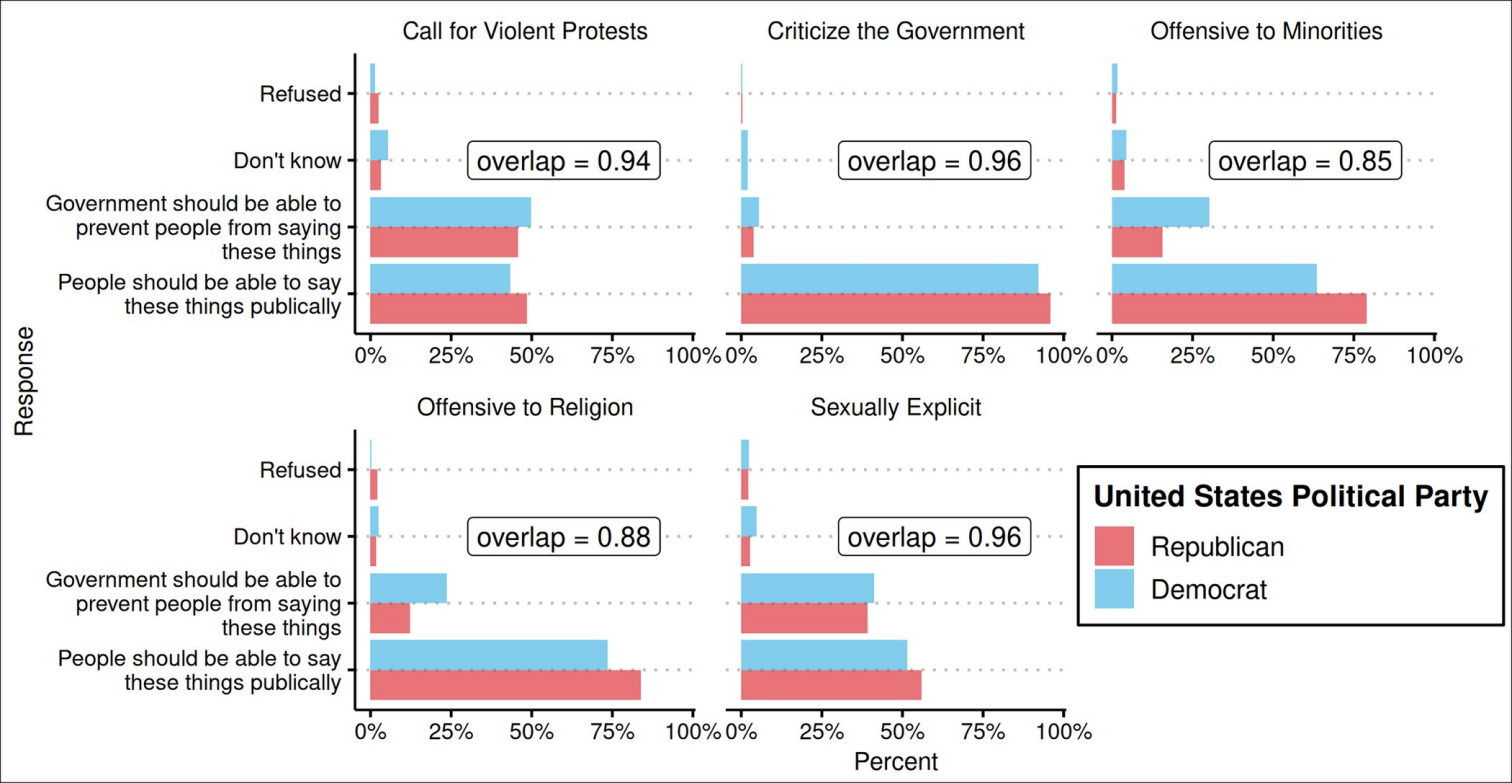
Democrat-Republican overlap for freedom of speech issues.

**Fig 6:**
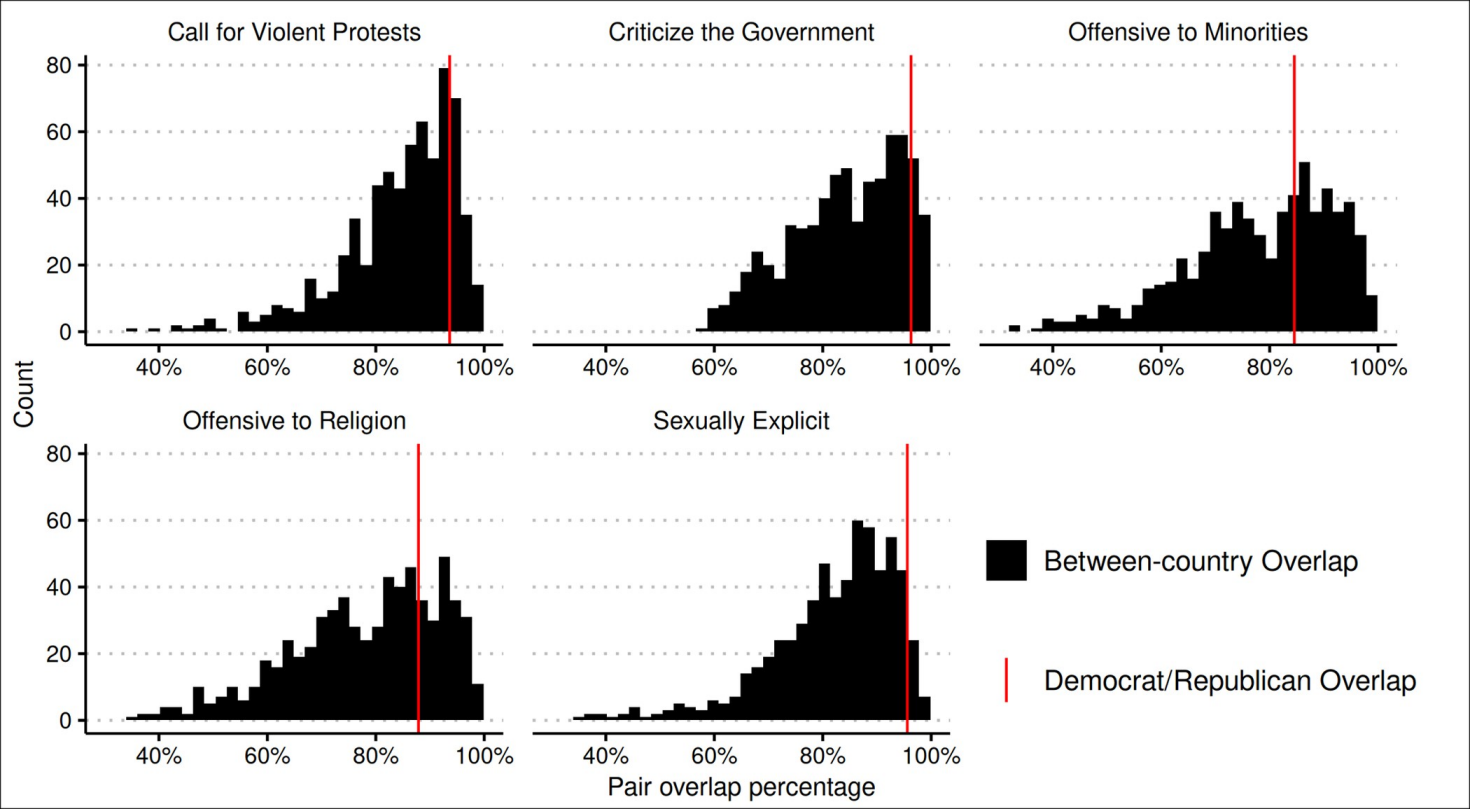
Distribution of all country pair overlap for freedom of speech issues.

As with the moral issues dataset, we first calculated the Democrat-Republican overlap coefficient for each issue of free speech along with bootstrapped 95% confidence intervals for all overlap coefficients (see Fig 5), and the overlap coefficients for all country pairs on the same five issues (see Fig 6). Finally, for each issue, we calculated the percentage of country pair overlaps that were smaller than the Democrat-Republican overlap, along with bootstrapped 95% confidence intervals for those statistics (see Table 2).

**Table 2:** Overlap coefficients for freedom of speech issues.

	Democrat-Republican Overlap			The Democrat-Republican overlap is greater than X% of the country pairs		
Freedom of Speech Issues	Overlap Coefficient	95% Confidence Interval of Overlap Coefficient		X Statistic	95% Confidence Interval of X statistic	
Call for Violent Protests	94%	89%	100%	82%	65%	100%
Criticize the Government	96%	94%	100%	88%	79%	100%
Offensive to Minorities	85%	78%	92%	60%	40%	77%
Offensive to Religion	88%	82%	95%	72%	54%	91%
Sexually Explicit	96%	93%	100%	94%	89%	100%

The overall pattern of results is similar to that of the moral issues. First, Democrats and Republicans showed some evidence of disagreement: the Democrat-Republican overlap coefficients for all issues are at 85% or above, and on average showed 92% overlap, but none reached 100%. However, putting Democrat-Republican disagreement in global context revealed that, across the five freedom of speech issues, the average Democrat-Republic overlap is greater than 79% of the country pair overlaps. Indeed, for all five freedom of speech issues, the Democrat-Republic overlap was greater than more than half of the country pair overlaps as the confidence intervals for those statistics excluded 50% (columns 5–6 in Table 2). These results for free speech issues are consistent with the pattern observed on moral issues.

## Discussion

In conclusion, our results clearly demonstrate differences between Republicans and Democrats. An overlap of just 66% in beliefs about the moral acceptability of abortion is no small divide to bridge when forming public policy; relatively small differences remain differences nonetheless, and it is clear that American politics is polarized. However, when reframed and viewed in global context, our results raise the possibility that–for both moral issues and freedom of speech–Democrats and Republicans may share more of a common set of beliefs than is typically believed. We suggest that such cross-cultural comparisons are a generally useful tool for understanding the magnitude of gaps in partisan perception. While we focused on comparing political groups within the United States to other countries–primarily because these data were most readily available–the same strategy can be used to further examine within- versus between-country similarities and differences around the world. Moreover, building on recent research showing that emphasizing similarities leads to more accurate lay perceptions [30–31] and reduces intergroup conflict [35–36]–placing within-country differences in global context could have the potential to offer a novel strategy for decreasing perceptions of difference.

## Supporting information

S1 Appendix section 1 Supporting information for dataset with moral issues. (DOCX)

S2 Appendix Section 2 Supporting information for dataset with freedom of speech issues. (DOCX)

S1 Table Percentage of respondents across options for eight moral issues for Democrats, Republicans, and Independents in the United States. (DOCX)

S2 Table Percentage of respondents across options for five freedom of speech statements for Democrats, Republicans, and Independents in the United States. (DOCX)
