# Supplementary material for: Putting Within-Country Political Differences in (Global) Perspective
Source: PLoS One. 2020 Apr 23;15(4):e0231794. doi: 10.1371/journal.pone.0231794 (PMC7179846; doi:10.1371/journal.pone.0231794)
Supplement: S1 Appendix section 1 — (DOCX) [file pone.0231794.s001.docx]

**SI Appendix Section 1: Supporting information for dataset with moral issues**

*Morality Items*

1. Do you personally believe that having an abortion is morally acceptable, morally unacceptable, or is it not a moral issue?
2. Do you personally believe that drinking alcohol is morally acceptable, morally unacceptable, or is it not a moral issue?
3. Do you personally believe that using contraceptives is morally acceptable, morally unacceptable, or is it not a moral issue?
4. Do you personally believe that getting a divorce is morally acceptable, morally unacceptable, or is it not a moral issue?
5. Do you personally believe that married people having an affair is morally acceptable, morally unacceptable, or is it not a moral issue?
6. Do you personally believe that gambling is morally acceptable, morally unacceptable, or is it not a moral issue?
7. Do you personally believe that homosexuality is morally acceptable, morally unacceptable, or is it not a moral issue?
8. Do you personally believe that sex between unmarried adults is morally acceptable, morally unacceptable, or is it not a moral issue?

*Response Options for Morality Items*

- Morally acceptable
- Morally unacceptable
- Not a moral issue
- Depends on the situation
- Don’t know
