# Supplementary material for: Putting Within-Country Political Differences in (Global) Perspective
Source: PLoS One. 2020 Apr 23;15(4):e0231794. doi: 10.1371/journal.pone.0231794 (PMC7179846; doi:10.1371/journal.pone.0231794)
Supplement: S2 Appendix Section 2 — (DOCX) [file pone.0231794.s002.docx]

**SI Appendix Section 2: Supporting information for dataset with freedom of speech issues**

*Freedom of Speech Items*

1. Do you think that people should be able to say these types of things publically OR that the government should be able to prevent people from saying these types of things in some circumstances? Statements that criticize the government's policies
2. Do you think that people should be able to say these types of things publically OR that the government should be able to prevent people from saying these types of things in some circumstances? Statements that are offensive to minority groups
3. Do you think that people should be able to say these types of things publically OR that the government should be able to prevent people from saying these types of things in some circumstances? Statements that are offensive to your religion or beliefs
4. Do you think that people should be able to say these types of things publically OR that the government should be able to prevent people from saying these types of things in some circumstances? Statements that call for violent protests
5. Do you think that people should be able to say these types of things publically OR that the government should be able to prevent people from saying these types of things in some circumstances? Statements that are sexually explicit

*Response Options for Freedom of Speech Items*

- People should be able to say these things publically
- Government should be able to prevent people from saying these things
- Don’t know
- Refused
