# Supplementary material for: Putting Within-Country Political Differences in (Global) Perspective
Source: PLoS One. 2020 Apr 23;15(4):e0231794. doi: 10.1371/journal.pone.0231794 (PMC7179846; doi:10.1371/journal.pone.0231794)
Supplement: S1 Table — (DOCX) [file pone.0231794.s003.docx]

**S1 Table. Percentage of respondents across options for eight moral issues for Democrats, Republicans, and Independents in the United States**

|  | Abortion | Alcohol use | Contraception use | Divorce | Extramarital Affairs | Gambling | Homosexuality | Premarital Sex |
| --- | --- | --- | --- | --- | --- | --- | --- | --- |
| U.S. Democrats |  |  |  |  |  |  |  |  |
| Morally acceptable | 22.9 | 31.4 | 50.0 | 37.5 | 4.3 | 26.2 | 25.6 | 31.4 |
| Morally unacceptable | 34.5 | 17.4 | 4.9 | 14.0 | 76.8 | 22.3 | 29.0 | 23.8 |
| Not a moral issue | 32.0 | 47.0 | 39.9 | 41.2 | 15.5 | 48.2 | 39.6 | 41.8 |
| Depends on the situation | 8.5 | 4.0 | 2.1 | 5.8 | 0.9 | 1.5 | 1.2 | 1.5 |
| Don’t know | 0.6 | 0.3 | 2.1 | 0.9 | 0.6 | 1.8 | 2.1 | 0.6 |
| Refused | 1.5 | 0.0 | 0.9 | 0.6 | 1.8 | 0.0 | 2.4 | 0.9 |
| U.S. Republicans |  |  |  |  |  |  |  |  |
| Morally acceptable | 8.5 | 35.1 | 53.3 | 29.3 | 1.9 | 24.3 | 17.4 | 25.1 |
| Morally unacceptable | 67.2 | 17.8 | 7.3 | 35.1 | 92.3 | 28.6 | 53.7 | 49.0 |
| Not a moral issue | 15.1 | 39.8 | 34.7 | 25.9 | 3.9 | 42.1 | 23.2 | 20.5 |
| Depends on the situation | 6.6 | 4.2 | 0.4 | 6.6 | 0.8 | 2.7 | 0.8 | 1.2 |
| Don’t know | 1.5 | 1.9 | 2.3 | 1.2 | 0.4 | 1.5 | 2.3 | 2.3 |
| Refused | 1.2 | 1.2 | 1.9 | 1.9 | 0.8 | 0.8 | 2.7 | 1.9 |
| U.S. Independents |  |  |  |  |  |  |  |  |
| Morally acceptable | 19.8 | 34.4 | 51.0 | 34.1 | 2.9 | 25.4 | 19.2 | 26.2 |
| Morally unacceptable | 43.7 | 12.0 | 6.7 | 18.4 | 85.1 | 20.1 | 34.1 | 28.9 |
| Not a moral issue | 27.4 | 49.0 | 37.6 | 40.8 | 10.8 | 52.2 | 42.6 | 41.7 |
| Depends on the situation | 6.4 | 3.2 | 1.2 | 5.0 | 0.3 | 0.6 | 0.3 | 1.2 |
| Don’t know | 1.5 | 1.2 | 2.3 | 1.5 | 0.3 | 0.9 | 2.0 | 1.2 |
| Refused | 1.2 | 0.3 | 1.2 | 0.3 | 0.6 | 0.9 | 1.7 | 0.9 |

Source: Pew Research Center’s Global Attitudes Spring 2013 Survey Data
