# Supplementary material for: Putting Within-Country Political Differences in (Global) Perspective
Source: PLoS One. 2020 Apr 23;15(4):e0231794. doi: 10.1371/journal.pone.0231794 (PMC7179846; doi:10.1371/journal.pone.0231794)
Supplement: S2 Table — (DOCX) [file pone.0231794.s004.docx]

**S2 Table. Percentage of respondents across options for five freedom of speech statements for Democrats, Republicans, and Independents in the United States**

|  | Call for violent protests | Criticize the government | Offensive to minority groups | Offensive to your religion and beliefs | Sexually explicit |
| --- | --- | --- | --- | --- | --- |
| U.S. Democrats |  |  |  |  |  |
| People should be able to say these things publically | 43.3 | 92.1 | 63.6 | 73.5 | 51.5 |
| Government should be able to prevent people from saying these things | 49.8 | 5.5 | 30.2 | 23.7 | 41.2 |
| Don't know | 5.5 | 2.1 | 4.5 | 2.4 | 4.8 |
| Refused | 1.4 | 0.3 | 1.7 | 0.3 | 2.4 |
| U.S. Republicans |  |  |  |  |  |
| People should be able to say these things publically | 48.6 | 95.8 | 79.0 | 83.9 | 55.9 |
| Government should be able to prevent people from saying these things | 45.8 | 3.8 | 15.7 | 12.2 | 39.2 |
| Don't know | 3.1 | 0.0 | 3.8 | 1.7 | 2.8 |
| Refused | 2.4 | 0.3 | 1.4 | 2.1 | 2.1 |
| U.S. Independents |  |  |  |  |  |
| People should be able to say these things publically | 42.2 | 98.6 | 74.5 | 84.1 | 55.3 |
| Government should be able to prevent people from saying these things | 52.6 | 0.5 | 21.1 | 13.2 | 40.8 |
| Don't know | 2.7 | 0.5 | 2.5 | 1.4 | 2.7 |
| Refused | 2.5 | 0.3 | 1.9 | 1.4 | 1.1 |

Source: Pew Research Center’s Global Attitudes Spring 2015 Survey Data
